# The *In Vitro* Effect of Psoralen on Glioma Based on Network Pharmacology and Potential Target Research

**DOI:** 10.1155/2022/1952891

**Published:** 2022-08-27

**Authors:** Yang Wu, Yong-Zheng Zhang, Meng-Jia Li, Wen-qing Yang, Lu-feng Cheng

**Affiliations:** Xinjiang Medical University, Urumqi, Xinjiang, 830011, China

## Abstract

Glioma is an aggressive tumor, currently there is no satisfactory management available. Psoralen, as a natural product, has been found to have an effect of treating cancer in recent years, but its effect on glioma has not been explored. In this study, we investigated the in vitro inhibition effect and potential targets of psoralen on glioma through network pharmacology and in *vitro* glioma treatment experiments. First, we used network pharmacology to preliminarily predict the 21 core genes of psoralen in the treatment of glioma, including PIK3CA, PIK3CB, PIK3CG, and JAK2. The CCK-8 method was used to detect the effect of psoralen on the proliferation of glioma U87 and U251 cells, and the results showed that psoralen could significantly inhibit the proliferation of U87 and U251 cells. The flow cytometry was used to detect the apoptosis and cell cycle changes, and it was found that psoralen could significantly promote the early apoptosis of U87 and U251 cells and had a significant cycle arrest effect on the two cells. The cell scratch test showed that psoralen could significantly inhibit the migration of U87 and U251 cells. The relative expression levels of PIK3CA, PIK3CB, PIK3CG, and JAK2 were analyzed by Real-time Quantitative polymerase chain reaction (QT-PCR), and the results showed that psoralen could inhibit the gene expression of PIK3CA, PIK3CB, PIK3CG, and JAK2. Later, Western blotting (WB) experiments showed that psoralen could inhibit the protein expressions of PI3K and JAK2. This study has preliminarily explored and verified the antiglioma effect of psoralen in the form of inhibiting cell proliferation and migration, promoting cell apoptosis and organizing cell cycle in vitro. And may play a role by inhibiting the expression of PIK3CA, PIK3CB, PIK3CG, JAK2 gene and PI3K, JAK2 protein, psoralen has become a potential antiglioma drug.

## 1. Introduction

Glioma is the most common primary brain tumor in adults. It is divided into I, II, III and IV grades by the World Health Organization [1], The higher the grade, the more serious the malignancy. Every year, about 100,000 people worldwide are diagnosed with diffused glioma [2], diffused glioma accounts for less than 1% of all newly diagnosed cancers, it is extremely high mortality rate compared with other cancers cannot be underestimated [3, 4]. Almost all high-grade gliomas recur, and no safe and less-toxic treatment has been reported [5].

In recent years, it has been found that the anti-cancer active components in plants have low, toxic and side effects while Therapeutically active, so they have good clinical application value. The furocoumarin psoralen is present in various natural plant, such as fructus psoraleae, fructus citri limoniae, and fructus fici of xinjiang indigenous medicinal plants [6], it has the characteristics of strong pharmacological activity, low toxicity, good bioavailability and good curative effect [7]. However, the research on psoralen in the treatment of glioma is still unexplored.

In this study, network pharmacology was used to preliminarily predict the potential targets of psoralen for the treatment of glioma. It was verified by pharmacodynamic experiments such as cell proliferation, cell apoptosis, cell cycle, and cell migration, and the predicted target genes were quantitatively analyzed to provide a scientific basis for further mechanism research.

## 2. Materials and Methods

### 2.1. Prediction of Key Targets by the Network Pharmacology

Description, sdf structure and all the related information were obtained from pubchem database (https://pubchem.ncbi.nlm.nih.gov/) and uploaded to SwissTargetPrediction database (http://www.swisstargeting.ch) for psoralen target search. Glioma disease targets were searched in OMIM database (https://omim.org/), Genecards database (https://www.genecards.org/) and CTD database (https://ctdbase.com/). Drugs and disease targets were intersected on the platform of the Venny2.1 online software (https://bioinfogp.cnb.csic.es/tools/venny/index.html) mapping tool to draw the Venn diagram. Drug-disease common targets were put into a STRING database (https://cn.string-db.org) to construct a PPI network for protein interaction. The data of protein-protein interaction network relationships in the STRING database was imported into Cytoscape software. The stress, radiosity, MNC, MCC, degree, eccentricity, closeness, and EPC methods of Cytohubba in Cytoscape was used to screen the core genes, and the number of intersecting genes were displayed by using the update function in R3.6.3 Gene coexpression and functional analysis of the core genes were performed using GeneMania (https://GeneMania.org).

### 2.2. Cell Culture

U87 and U251 cell line were purchased from Shanghai Zhongqiao Xinzhou Biotechnology. U87 and U251 cells were cultured in DMEM containing 10% fetal bovine serum (FBS, Gibco) and 1% penicillin/streptomycin (Hyclone, USA) and placed in an incubator at 37°C with 5% CO_2_ for routine culture. The cell culture medium was changed once every two days. The cells in the logarithmic phase were taken for experiments.

### 2.3. Cell Proliferation Detected by the CCK-8 Assay

U87 and U251 cells were seeded into 96-well plates at a density of 4 × 10^3^/wells. Cells were treated with various concentrations of psoralen (MCE, USA) (0 *μ*M, 5 *μ*M, 10 *μ*M, 20 *μ*M, 40 *μ*M, and 80 *μ*M). After cell incubation for 24 h and 48 h, 10 *μ*L of CCK-8 (Biosharp, CHN) was added into each well and incubated for 1 h. The optical density (OD) rate was measured at 450 nm by using a microplate reader. The cell inhibition rate was calculated according to the following formula:

(1) $$\begin{array}{c} \text{inhibition rate}\left(\%\right)=\frac{0\mu \text{MOD}-\text{psoralen OD}}{0\mu \text{MOD}-\text{blank OD}}\times 100\%, \end{array}$$

The semi-inhibitory concentration (IC_50_) was calculated using Graphpad prism 8.

### 2.4. Cell Apoptosis Detected by the Flow Cytometry Assay

U87 and U251 cells were seeded into 6-well plates at a density of 2 × 10^5^/wells. The cells were cultured for 12 hours to adhere. The medium was removed and psoralen (0 *μ*M, 10 *μ*M, and 30 *μ*M) was administered. Then the cells were collected by trypsin (Hyclone, USA) digestion after 24 h. Cells were washed with precooled Phosphate Buffered Saline (PBS) and blended in 100 *μ*L·l × binding buffer, where 5 *μ*L Annexin-V and 5 *μ*L7-AAD (BD, USA) were added. The cells were incubated in the dark for 15 min at room temperature (RT) and blended in 400 *μ*L·1 × binding buffer, detected by flow cytometry.

### 2.5. Cell Cycle Detected by the Flow Cytometry Assay

U87 and U251 cells were seeded into 6-well plates at a density of 5 × 10^6^/mL. The cells were cultured for 12 hours to adhere. The medium was removed and psoralen (0 *μ*M, 10 *μ*M, and 30 *μ*M) was administered. After 48 h, the cells were washed with precooled PBS, digested by trypsin to collect cells, washed with PBS again, blended cells in precooled 70% ethanol at 4°C overnight. On the next day, after being washed twice, 500 *μ*L PI/RNase (BD, USA) was added for staining, incubated in the dark for 15 min at RT, and detected by flow cytometry.

### 2.6. Cell Migration Detected by the Wound Healing Assay

U87 and U251 cells were seeded into 6-well plates at a density of 2 × 10^5^/mL using the cell scratch test. After attachment for 12 h of culture, a 200 mL pipette tip was used to mark several scratches at intervals of about 10 mm, and psoralen (0 *μ*M, 10 *μ*M, and 30 *μ*M) was administered after cell debris was washed out with PBS. Three fields of view were randomly selected from each well, and the scratches were photographed at 0 h, 24 h, and 48 h. Image-J software was used to analyze the gray value of the scratch. The healing rate was calculated according to the following formula:

(2) $$\begin{array}{c} \text{healing rate}\left(\%\right)=\frac{0\text{ h scratch distance}-24/48\text{ scratch distance}}{0\text{ h scratch distance}}. \end{array}$$

### 2.7. Expression of mRNA Detected by the RT-PCR Assay

The total RNA in U87 and U251 cells of the 0 *μ*M, 10 *μ*M, and 30 *μ*M psoralen dosing groups was extracted with TRIZOL (ambion, USA) reagent. The purity and content of the total RNA was detected by an ultraviolet spectrophotometer. The extracted RNA was reverse transcribed into cDNA using the PrimeScriptTM 1st strand cDNA Synthesis Kit (TaKaRa, Japan). After the experiments were completed using the StepOnePlus real-time PCR system, the data were analyzed by using the 2^-△△Ct^ method. Primer sequences are listed in Table 1.

### 2.8. Expression of Protein Detected by the WB Assay

The total protein in U87 and U251 cells of psoralen (0 *μ*M, 10 *μ*M, and 30 *μ*M) was extracted with RIPA (Thermo, USA). The total protein content was determined by the BCA Protein Assay Kit (Thermo, USA). Proteins were separated by 10% SDS-polyacrylamide gel electrophoresis, transferred to PVDF membrane by Sandwich method, blocked by 5% defatted milk powder at RT for 1 h, and incubated with primary antibody at 4°C overnight. The PVDF membrane was washed 3 times with (TBS + Tween) TBST, incubated horseradish peroxidase labeled goat antirabbit IgG for 2 h, and washed 3 times with TBST. After the enhanced chemiluminescence (ECL) was added, it was exposed in an exposure apparatus, and the gray value of protein bands was analyzed by the Image-J image analysis system. WB antibodies were PI3K (Proteintech, 20584-1-AP), JKA2 (Abcam, ab108596), and *β*-actin (Abcam, ab8226).

### 2.9. Statistical Analysis

Statistical analysis was performed using SPSS 17.0 and GraphPad Prism 8. Measurement data were expressed as$\bar{x}\pm s$, and Student's *t*-test was used for comparison between two groups, and one-way analysis of variance was used for comparison among multiple groups. Where *P*^*∗*^ < 0.05 and *P*^*∗∗*^ < 0.01 indicated statistically significant difference.

## 3. Result

### 3.1. Network Pharmacology Prediction of Psoralen in Treatment of Glioma

**Figure 1:**
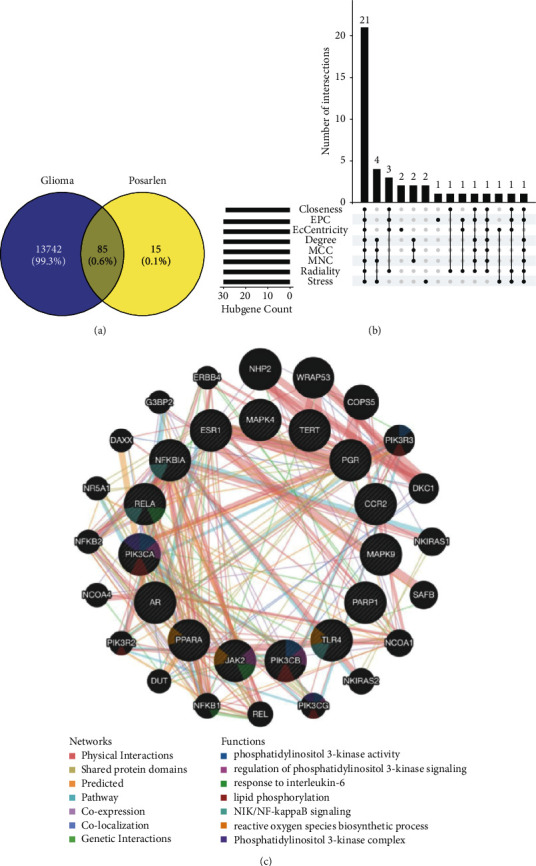
Network pharmacology prediction of psoralen in the treatment of gliomas. (a) Venn diagram, the intersection target of glioma and psoralen. (b) Predict a core gene upset map by eight algorithms. (c) Functional prediction of core gene.

A total of 102 potential psoralen targets were predicted by the SwissTargetPrediction database, and 13,827 glioma targets were predicted by the OMIM, Genecards, and CTD databases. A total of 85 intersection targets were collected after intersection via the Veeny website (Figure 1(a)). The first 30 core genes were screened using the eight algorithms of Cytohubba in Cytoscape, and the number of intersecting genes was analyzed using the Upset function in R3.6.3 (Figure 1(b)). Finally, 21 genes, including PIK3CA, JAK2, MAPK4, and PIK3CD were obtained (Figure 1(c)). Further analysis of the core genes revealed that PIK3CA, PIK3CB, JAK2, and RELA genes were involved in the regulation of phosphatidylinositol 3- kinase signal and interleukin-6 regulation.

### 3.2. Psoralen Inhibits the Proliferation of U87 Cells and U251 Cells

**Figure 2:**
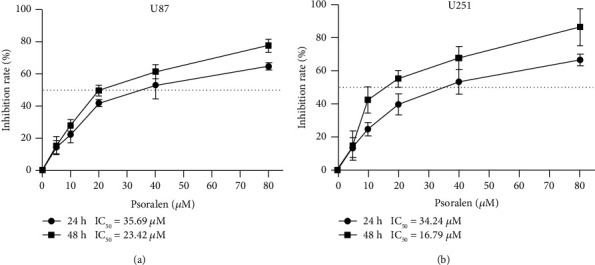
Psoralen can significantly inhibit the proliferation of U87 cells and U251 cells. The U87 cells and U251 cells were incubated with psoralen of different concentrations for 24 h/48 h, and then the cell proliferation was detected by CCK-8 assay. (a) U87 cells for 24 h (F = 177.91, (*P* < 0.01), half maximum inhibition concentration (IC50) = 35.69 *μ*M. 48 h (F = 141.54, *P* < 0.01), IC50 = 23.42 *μ*M. (b) U251 cells for 24 h (F = 206.56, (*P* < 0.01), IC50 = 34.24 *μ*M. 48 h (F = 65.93, (*P* < 0.01), IC_50_ = 16.79 *μ*M). There were 5 secondary wells for each sample and each experiment was conducted in triplicate.

Psoralen significantly inhibits the proliferation of U87 and U251 cells after 24 h and 48 h of administration of 0 *μ*M, 5 *μ*M, 10 *μ*M, 20 *μ*M, 40 *μ*M, and 80 *μ*M psoralen, and the inhibition capacity increased with the increase of psoralen concentration in a significant concentration-dependent manner. The inhibition rates had significant statistical differences among dosage groups, as shown in Figure 2.

### 3.3. Psoralen Can Significantly Promote the Apoptosis of U87 Cells and U251 Cells

**Figure 3:**
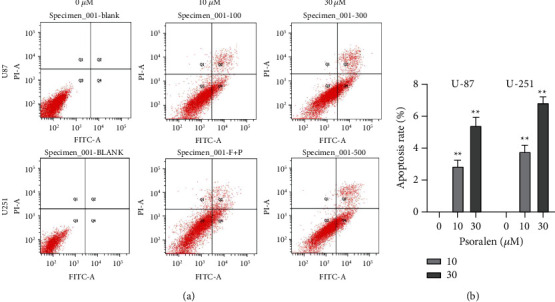
Psoralen can significantly promote the apoptosis of U87 cells and U251 cells. The U87 cells and U251 cells were incubated with psoralen (0 *μ*M, 10 *μ*M, and 30 *μ*M) for 12 h then the cell apoptosis was detected by flow cytometry assay. (a) Flow cytometry scatter plot of psoralen (0 *μ*M, 10 *μ*M, and 30 *μ*M) given to U87 and U251 cells. (b) Compared with 0 *μ*M, psoralen of 10 *μ*M and 30 *μ*M can significantly promote the apoptosis of U87 and U251 cells, ^∗^*P* < 0.01. There were 3 secondary wells for each sample and each experiment was conducted in triplicate.

The results of Annexin V-FITC/PI double staining for the detection of apoptosis in U87 and U251 cells showed that after the administration of 10 *μ*M and 30 *μ*M psoralen, psoralen significantly promoted the early apoptosis of these two cells, and this ability was increased with the increase of psoralen concentration in a significant concentration-dependent manner. The inhibition rates had significant statistical differences between the dosage groups (Figure 3).

### 3.4. Psoralen Can Significantly Arrest the Cell Cycle of U87 and U251 Cells

**Figure 4:**
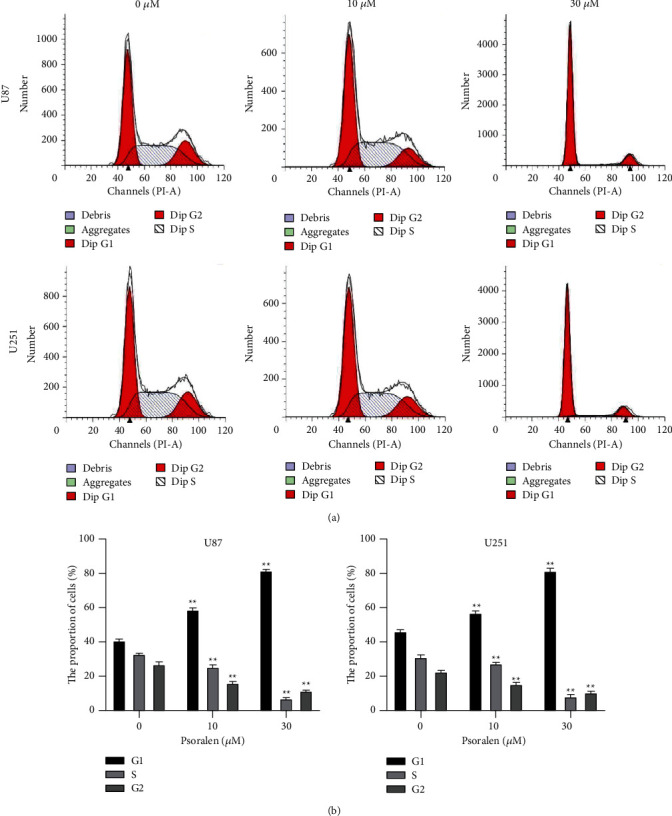
Psoralen blocks the cell cycle. The U87 cells and U251 cells were incubated with psoralen (0 *μ*M, 10 *μ*M, and 30 *μ*M) for 12 h then the cell cycle was detected by flow cytometry assay. (a) Flow cytometry of cell cycle after administration of U87 and U251 cell psoralen (0 *μ*M, 10 *μ*M, and 30 *μ*M). (b) The G1 phase of U87 cells and U251 cells after administration of psoralen (10 *μ*M, 30 *μ*M) was significantly higher than the 0 *μ*M psoralen group, ^∗^*P* < 0.01. S phase and G2 phase of two cells after administration of psoralen (10 *μ*M, 30 *μ*M) were significantly lower than the 0 *μ*M psoralen group, ^∗^*P* < 0.01. There were 3 secondary wells for each sample, and each experiment was conducted in triplicate.

The cell cycle results of U87 and U251 by flow cytometry showed that after administration of 10 *μ*M and 30 *μ*M psoralen, the proportion of cells in G1 phase was increased while that in S phase and G2 phase was decreased due to psoralen, showing a significant concentration dependence. The inhibition rates showed significant statistical differences among the dosage groups (Figure 4). It indicated that psoralen arrests the cells in the G1 phase.

### 3.5. Psoralen Can Significantly Reduce the Migration Ability of U87 Cells and U251 Cells

**Figure 5:**
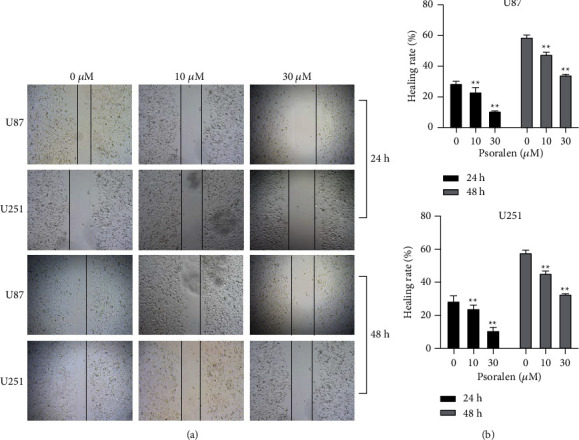
Psoralen can significantly reduce the migration ability of U87 cells and U251 cells. The U87 cells and U251 cells were incubated with psoralen (0 *μ*M, 10 *μ*M, and 30 *μ*M) for 24 h/48 h Then that healing rate is observed under a microscope. (a) Cell wound pictures of U87 cells and U251 cells. At 24 h and 48 h after psoralen (0 *μ*M, 10 *μ*M, and 30 *μ*M) administration. (b) The healing rate of U87 cells and U251 cells after administration of psoralen (10 *μ*M, 30 *μ*M) was significantly lower than the 0 *μ*M psoralen group, ^∗^*P* <0.01. Take 3 fields of view per group and each experiment was conducted in triplicate.

The wound healing assay results showed that psoralen can significantly inhibit the migration ability of U87 and U251 cells under 24 h and 48 h photographing after administration of 10 *μ*M and 30 *μ*M psoralen. The effects were significant in the 10 *μ*M and 30 *μ*M groups compared with the 0 *μ*M group (Figure 5).

### 3.6. Psoralen Decreased PIK3CA, PIK3CB, PIK3CG, and JAK2 Relative Gene Expression

**Figure 6:**
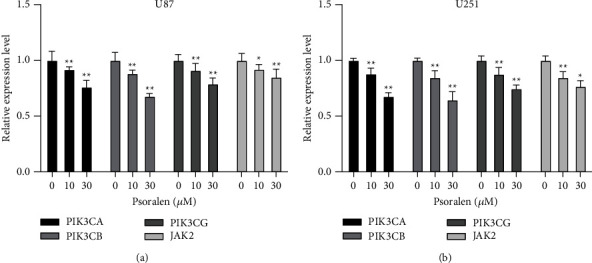
Psoralen can significantly reduce the expression of PIK3CA, PIK3CB, PIK3CG, and JAK2. The U87 cells and U251 cells were incubated with psoralen (0 *μ*M, 10 *μ*M, and 30 *μ*M) for 24 h then RNA was extract, and that relative expression level of the gene was detected by RT-PCR assay. (a) The PIK3CA, PIK3CB, PIK3CG, and JAK2 expression levels of psoralen (10 *μ*M, 30 *μ*M) were both decreased as compared with 0 *μ*M psoralen in U87 cells, ^∗^*P* < 0.01, ^∗∗^*P* < 0.05. (b) The PIK3CA, PIK3CB, PIK3CG, and JAK2 expression levels of psoralen (10 *μ*M, 30 *μ*M) were both decreased as compared with 0 *μ*M psoralen in U251 cells, ^∗^*P* <0.01, ^∗∗^*P* < 0.05. There were 5 secondary wells for each sample and each experiment was conducted in triplicate.

The results of RT-PCR experiments showed that after the two cells were given 10 *μ*M and 30 *μ*M psoralen, the relative gene expression levels of PIK3CA, PIK3CB, PIK3CG, and JAK2 were decreased, and the inhibition ability increased in a concentration-dependent manner with the increase in concentration. The gene expression levels in the 10 *μ*M and 30 *μ*M groups were significantly different from those in the 0 *μ*M group (Figure 6).

### 3.7. Psoralen Decreased PI3K and JAK2 Protein Expression

**Figure 7:**
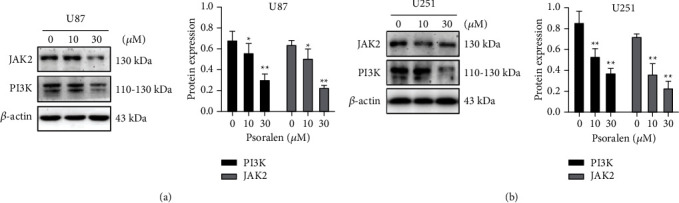
Psoralen can significantly reduce the expression of PIK3CA, PIK3CB, PIK3CG, and JAK2. The U87 cells and U251 cells were incubated with psoralen (0 *μ*M, 10 *μ*M, and 30 *μ*M) for 24 h then protein was extracted, and the relative expression level of the gene was detected by WB assay. (a) The JAK2 and PI3K protein levels of psoralen (10 *μ*M, 30 *μ*M) were both decreased as compared with 0 *μ*M psoralen in U87 cells, *∗P* < 0.01, ^∗∗^*P* < 0.05. (b) The JAK2 and PI3K protein levels of psoralen (10 *μ*M, 30 *μ*M) were both decreased as compared with 0 *μ*M psoralen in U87 cells, ^∗∗^*P* < 0.05. Each experiment was conducted in triplicate.

The results of WB experiments showed that after the two cells were given 0 *μ*M, 10 *μ*M, and 30 *μ*M psoralen. Protein expression levels of PI3K and JAK2 were decreased, and the inhibition ability increased in a concentration-dependent manner with the increase in concentration. The protein expression levels in the 10 *μ*M and 30 *μ*M groups were significantly different from those in the 0 *μ*M group. The inhibition effects of 30 *μ*M psoralen on the two proteins expression were significantly stronger than those of the positive drugs (Figure 7).

## 4. Discussion

Glioma is one of the most aggressive solid tumors among all intracranial tumors. Due to its complex pathogenesis, the strong toxicity of conventional chemotherapy drugs and the easy development of drug resistance in tumor cells, it brings difficulties to the treatment and prognosis of glioma. This is mainly because the genome of glioma has a high degree of mutation, which is involved in the regulation of multiple key signaling pathways such as cell growth, proliferation, survival, and apoptosis.

Psoralen, as a natural product, has good anti-inflammatory and anticancer effects. Wang Xiaohong [8] found that psoralen induced breast cancer cell cycle block by regulating the Wnt/*β*-catenin pathway, and there was no significant toxicity at the effective concentration. At the same time, psoralen also plays an important role in reversing multi-drug resistance in breast cancer [9]. Methoxsalen, a psoralen derivative, has been shown to significantly inhibit the activity of rat glioma C6 cells, normal astrocytes, and human glioblastoma GL-15 cells in combination with UVA *in vitro* [10], we conducted further research on the pharmacodynamic mechanism of psoralen on glioma.

A large number of studies have proved that the phosphate-3-kinase/protein kinase (PI3K/Akt) pathway has a tight correlation with glioma, and the pathway has been changed in 70% of glioma cells [11–13]. The activation of PI3K can significantly promote the survival, proliferation, invasion, and migration of cancer cells [14, 15]. There are four classes of PI3Ks (Classes IA, IB, II, and III) in mammals, including humans.

Class IA PI3K is composed of three homo-isomers: PIK3CA, PIK3CB, and PIK3CD encode the catalytic subunits p110*α*, p110*β*, and p110*δ*, respectively. These subunits combine with a p85 regulatory subunit (*α*, *β*, or *γ*) encoded by PIK3R1, PIK3R2, and PIK3R3 to form heterodimers. Class IB PI3K has a fourth catalytic isomer, PIK3CG (encoding p110*γ*), which is expressed in immune cells by interacting with a regulator p101 encoded by PIK3R5 [16].

The network pharmacology method was used to find that the three genes of PIK3CA, PIK3CB, and PIK3CG had a very high correlation with the treatment of glioma with psoralen. In the RT-PCR and WB experiments, the expression levels of PIK3CA, PIK3CB, PIK3CG, and PI3K protein were decreased by psoralen. PIK3CA has been shown to be one of the most frequently mutated genes in solid tumors that regulate cell proliferation, angiogenesis, growth, movement, and survival by involving the PI3K/Akt signaling pathway [17–19]. Relevant studies have found that the activation mutation of PIK3CA is related to the early recurrence and poor prognosis of glioblastoma. In addition, the mutation of PIK3CA significantly activates the PI3K pathway to increase the malignancy of gliomas and make them more prone to recurrence [20–22]. PIK3CB has been reported to be associated with a high recurrence rate of gliomas and also increases the risk of developing gliomas, resulting in decreased survival [23]. Restoring PTEN in glioblastoma cells or knocking down PIK3CB can significantly downregulate the phosphorylation of AKT, inhibit cell proliferation, block the cell cycle, and promote apoptosis in the G0/G1 phase [24]. At present, there is little research on PIK3CG, but it is found that in medulloblastoma, the overexpression of PIK3CG contributes to the proliferation of medulloblastoma cells and improves resistance to cisplatin therapy [25].

JAK2 was predicted to have decreased expression in network pharmacology. The correlation between the JAK2/STAT3 pathway and glioma is extremely high. Phosphorylation of JAK2 activates STAT3 expression and induces abnormal proliferation of glioma cells, which finally leads to further deterioration of the disease and endangers life [26–28]. In addition, some studies have pointed out that the JAK2/STAT3 pathway can act as an upstream immune signal to regulate the PI3K/AKT pathway and affect the therapeutic effect of glioma [29].

Based on previous literature and our study, we speculated that psoralen inhibited the proliferation and migration ability of tumor cells, promoted apoptosis, and blocked the cell cycle by reducing the expression levels of four genes: JAK2, PIK3CA, PIK3CB, and PIK3CG. Psoralen may be able to play a role as a potential inhibitor of JAK2 and PI3K in the treatment of glioma, but the data in this study is only limited to the effects of psoralen on two glioma cell lines in *vitro*, and further insights are required to explore the extensive experimental research in this domain.

## 5. Conclusion

In conclusion, this study found that psoralen has a good antiglioma pharmacological effect in *vitro*. These effects may be profoundly achieved by inhibiting the expression of JAK2, PIK3CA, PIK3CB, and PIK3CG genes and JAK2 and PI3K proteins, inhibiting the proliferation of cancer cells and promoting the apoptosis and migration of cancer cells. Psoralen has the ability to achieve cell cycle arrest of cancer cells at the G1 phase.

## Figures and Tables

**Table 1:** Primer sequences for the RT-PCR assay.

Genes	Sequences (5′ end to 3′ end)
PIK3CA	F: GAATAGGCAAGTCGAGGCAATG
	R: AGGGTTTAGAGGAGACAGAAAGC

PIK3CB	F: GCGACAGATGAGTGATGAAGAAC
	R: GCCCTATCCTCCGATTACCAAG

PIK3CG	F: GAATAGGCGACAGACACAATGAC
	R: GGTTAGCACAAATGGCACTCTC

JAK2	F: TCAGAGAAGAAGACAGGAAGACAG
	R: TTGAGAATCCAGAGCACTTAGAGG

GAPDH	F: CCCATCTATGAGGGTTACGC
	R: TTTAATGTCACGCACGATTTC

F, forward; R, reverse.

## Data Availability

The data used to support the findings of this study are available from the corresponding author upon request.
