# Correction to “The *In Vitro* Effect of Psoralen on Glioma Based on Network Pharmacology and Potential Target Research”

**DOI:** 10.1155/ecam/9831031

**Published:** 2025-12-22

Y. Wu, Y.‐Z. Zhang, M.‐J. Li, W. Yang, and L.‐F. Cheng, “The *In Vitro* Effect of Psoralen on Glioma Based on Network Pharmacology and Potential Target Research,” *Evidence-Based Complementary and Alternative Medicine* 2022, no. 1 (2022): 1–10, https://doi.org/10.1155/2022/1952891.

In the Results section, Figure 5a was incorrect. During the preparation of the manuscript, panels *0 μm U251, 10 μm U87, and 30 μm U251* were mistakenly uploaded in duplicate. The correct Figure 5 is shown below:

Figure 5:

**Figure:**
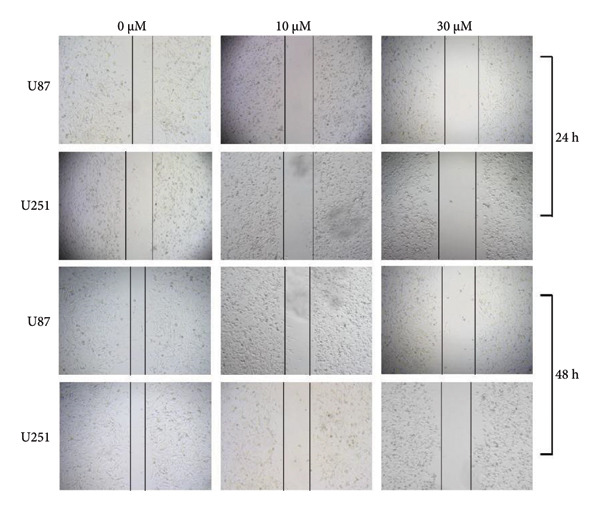
(a)

**Figure:**
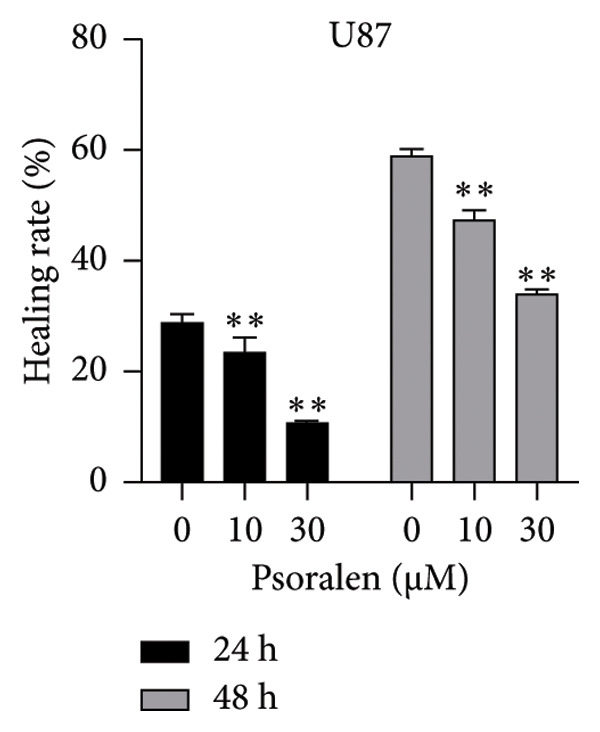
(b)

**Figure:**
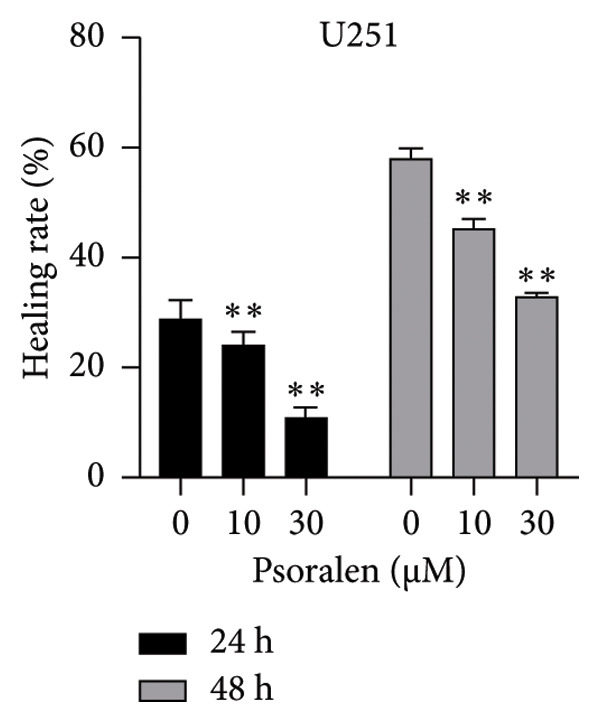
(c)

We apologize for this error.

